# 

**Published:** 1878-06

**Authors:** 


### INDEX TO VOLUME XXXVI.

Æ.

PAGE.

Æther and chloroform, death from ... 268

A.

Abortion, management of ... 414
Abscess—three cases of oral ... 89
Acne rosacea, treatment of ... 637
Aconite in tetanus ... 442
Advertising pictures ... 447
Allport, F., M. D., use of cold and heat in fevers ... 386
American Medical Association 555
Ametropia and Blepharitis, relations of, Dr. F. C. Hotz ... 367
Amyloid degeneration of liver, spleen and kidneys, case of .. 342
Anæsthesia, general ... 644
Anæsthesia, limited ... 652
Anæmia, associated with pregnancy, case ... 396
Anæsthetics in labor ... 413
Andrews, Edmund, lithotrity, cases of, etc ... 592
Announcements, monthly .. 112, 224 336, 448, 560, 672
Antiseptic dressing, Lister's ... 327
Antiseptic injections into joints 435
Anus, bismuth for prolapsus of 217
Aortic valves, digitalis in diseases of ... 74
Arkansas State Medical Society ... 527, 670
Arsenic in subnitrate of bismuth, Dr. J. H. Salisbury ... 601
Ascites, a case ... 164
Aspiration in acute pleurisy, M. Dieulafoy ... 436
Atrophy of the testicles after mumps ... 212

B.

Banga, Henry, M. D., cases by. 396
Bartlett, John, M. D., measurements of the extremities ... 484
Belfield, Dr. W. T., cases by ... 337 339, 342, 347, 351
Belfield, Wm. T., some observations on the clinical and pathological distinctions between phthisis and tuberculosis ... 1
Belladonna in spermatorrhœa . 274
Belladonna, tincture of, external use in night sweating ... 606
Bismuth for prolapsus ani and hæmorrhoids ... 217
Bismuth, sub-nitrate, arsenic in 601
Blackman, F. H., M. D., tracheotomy ... 381
Bladder, gunshot wound of, Dr. F. Staples ... 510
Bladder, treatment of catarrh of 80
Blanchard, Wallace, M. D., new apparatus for Pott's disease .. 376
Blepharitis and ametropia, relations of, Dr. F. C. Hotz ... 367
Blephorospasmus caused by excessive efforts of accommodation ... 278
Board of Health, Illinois State directory of ... 527
Board of Health, Michigan State 556
Boehm, R., resuscitation in poising and asphyxia ... 546
Bogue, R. G., tracheotomy in diphtheretic croup ... 121
Borax and nitrate of potassium in hoarseness ... 433
Breast, fibro-cystic tumor of ... 344
Brieger, Dr. L., action of cathartics ... 437
Bromide of potassium, treatment of eruption of ... 637
Brown-Séquard, Dr., original lectures paralysis and convulsions as effects of disease of the base of the brain. 291, 353, 449

PAGE.
Brown, Geo. E., case of abscess of spleen with perforation of large intestine.............. 15
Brown, S. A., M. D., remedy for eruption of poison oak, etc... 521
Brown-Séquard, review of recent lectures, by Dr. J. S. Jewell..................... 561
Burns, sudden death from..... 548
Byford, Wm. H., a case of extra uterine pregnancy.......... 113

C.

Caldwell, W. S., salicylate of soda in rheumatism, etc..... 56
Caldwell, W. S., M. D., letter from Vienna............... 417
Cancer of stomach and liver, case..................... 516
Carbolic acid, effect on color of urine.................... 643
Carcinoma, injection of acetic acid in................... 91
Carcinoma of intestine, case... 346
Carcinoma of tongue......... 47
Case, L. W., M. D............. 631
Cathartics, action of, Dr. L. Brieger.................... 437
Caton, Hon. J. D., statistics from Hawaiian Islands, by....... 613
Cephalotomy, cases...... .... 256
Cerium oxalate in chronic cough 642
Charcot, metallotherapy....... 642
Charities, medical, abuse of.... 606
Chicago Medical College, commencement................ 446
Children, art of prescribing for 331
Chloroform and æther, death from...................... 268
Chloroform, ingestion of six drachms, recovery.......... 398
Chloroform in tetanus......... 441
Chloral hydrate in tetanus..... 441
Chloral in retention of urine.. 542
Chorion, cystic degeneration of 176
Clark, Thomas, M. D.......... 642
Claude, Bernard, obituary..... 366
Cochlea, exfoliation of... .... 405
Cold and heat, use of in fevers, Dr. F. Allport.............. 386
Coloboma of iris, double congenital, cases............... 412
Concussion, pathology of...... 547
Conjunctiva, gummatous tumor of....................... 636
Contagious diseases, spread of.. 404
Convulsions and paralysis as effects of disease of the base of the brain, Dr. Brown-Séquard.............291, 353, 449

PAGE.
Copaiba and cubebs in croup.. 545
Coronary artery, thrombosis of 434
Cough, chronic, cerium oxalate in....................... 642
Couty, Dr.................. 644
Croup, copaiba and cubebs in, Dr. Oldoni................ 545
Croup membranous, cases, tracheotomy in................ 504
Crystalline lens, congenital displacement of.............. 554
Cutter, E., new stem pessary... 25
Cutter, E., ovarian tumor reported cured by galvanic current....................... 272

D.

Danforth, I. N., the pathological transactions of the Chicago Medical Society.... 1
Davis, N. S., M. D., purpura hæmorrhagica.............. 585
Dewey, Dr., case of carcinoma of intestine................ 346
Diabetes cured by skim-milk, Dr. H. W. Jones............ 31
Diabetes mellitus, liquorice in. 219
Diabetes mellitus, sodic salicylate in...................... 641
Diarrhœa, chlorate of potassium in........................ 220
Diarrhœa, injections of borate of sodium for............... 220
Dieulafoy, M., aspiration in acute pleurisy.............. 436
Digitalis in disease of aortic valves..................... 74
Digiti mortui, Dr. McBride... 643
Diphtheria benefited by inhalation of steam............... 275
Diphtheria and tracheotomy... 94
Diphtheria, tracheotomy in.329, 501
Diphtheria, treatment of...... 274
Diphtheritic croup, tracheotomy in....................... 121
Doering, E. J., M. D., clinical cases...................... 515
Dropsy, acute general, Dr. J. A. Goldsberry....... ........ 475
Duret M., pathology of consumption.................. 547
Dyas, W. Godfrey, lymphatic system.................... 279

E.

Eclampsia, cases..........251, 256
Electricity, value of in paralysis, Dr. Wm. E. Reynolds....... 403
Epithelioma of skin.......... 639

PAGE.
Epley, F. W., partial extirpation of parotid gland ........... 277
Ergotine, hypodermic injections of, in treatment of fibroid tumor of uterus .............. 604
Ergotine, sub-cutaneous injections of, in metrorrhagica... 543
Erysipelas, carbolic acid by sub-cutaneous injection in treatment of .................... 239
Esmarch's bandage in reduction of hernia .............. 638
Extra-pericardial adhesions.... 544
Extra-uterine pregnancy, case.. 113, 179
Extra-uterine pregnancy, normal labor during ............... 84
Eye diseases, report of 565 cases of .......................... 38
Eye and ear infirmary ........ 110
Eye, mechanical injuries of the globe ........................ 12

F.

Face-presentation, case........ 246
Farrar, Saml. F., on Prof. Nussbaum's operation of nerve-stretching ................. 225
Farquharson, R., art of prescribing for children........ 331
Fever, feigned................ 545
Fevers, use of cold and heat in, Dr. Allport................ 386
Fibro-cystic tumor of breast, case....................... 344
Fibro-sarcoma of lower lid, operation for............... 284
Finger-nails, long, in Annam.. 465
Fitch, T. D., M. D., report on obstetrics ................... 413
Forceps, delivery by.......... 252
Forceps, discussion on application of..................... 288
Forceps, short obstetrical...... 499
Fox, Geo. H., M. D., molluscum contagiosum .............. 466
Fracture of patella, treatment of 639

G.

Galvanic current, ovarian tumor reported cured by........... 272
Gertin, M. H., M. D., the bath in typhoid fever............ 33
Glycerine in hæmorrhoids.... 642
Goitre, treatment by injections. 93
Goldsberry, J. A., M. D., acute general dropsy.............. 475
Gonorrhœa, chronic, treatment of ........................ 215

PAGE.
Gonorrhœa, gurjun balsam in.. 326
Gout, treatment by cold water douche ..................... 212
Gradle, H., M. D.............. 631
Gray, S. S., M. D., milk sickness of animals .................. 533
Gummatous tumor of conjunctiva...................... 636
Gynæcology and gynæcologists abroad..................... 417

H.

Hall, J. M., M. D., society reports by.................... 405
Hammer H., M. D., case of thrombosis of coronary artery 434
Hand, presentation of—cases... 175
Hand and cord, presentation cases ....................... 245
Harrington, H. L., on treatment of diphtheria and spermatorrhœa ..................... 274
Havre, the doctors strike...... 426
Hawaiian Islands, vital statistics of, Hon. J. D. Caton..... 613
Hayes, P. S., abstract of paper on pneumogastric nerves.... 282
Hayes, P. S., M. D., case of fibro-cystic tumor of breast....... 344
Heart clots ................. 535
Heart disease and embolism, case of.................... 284
Hemi-anæsthesia, alternate. ... 649
Hemi-hyperæsthesia........... 660
Hemorrhage, post partem, cases 250
Hemorrhagic diathesis, case... 525
Hemorrhoids and prolapsus ani, bismuth for................ 217
Hepatitis, acute, case of....... 337
Hermaphrodism, a case....... 167
Hernia intractable, reduction by Esmarch's bandage.......... 638
Hernia, treatment of.......... 623
Hoarseness, borox and nitrate of potassium...............219, 433
Holmes, E. L., M. D., case of hemorrhagic diathesis....... 525
Holmes, E. L., mechanical injuries of the globe of eye, with numerous pathological specimens................. 12
Hospital, Alexian Brothers, reports ..................... 392
Hospital, U. S. Marine, San Francisco, reports of cases.. 514
Hot Springs of Arkansas...... 308
Hotz, F. C., operation for fibro-sarcoma of lower lid........ 284
Hotz, F. C., blepharospasmus.. 278

PAGE.
Hotz, F. C., M. D., the relation of ametropia to blepharitis... 367
Hot water as a hæmostatic..... 414
Humphreys, L., cystic degeneration of the chorion.......... 176
Hyde, J. N., innocuity of certain physiological secretions in syphilis.................... 145
Hyde, J. Nevins, M. D., phenomena, etc., of sexual orgasm 572
Hydrobromate of quinine, in diseases of children, Dr. Steinitz..................... 438
Hyperæsthesia, general........ 657
Hypodermic injections of morphia, danger from, Dr. E. Fletcher Ingals.............. 491
Hymen, imperforate, case...... 166

I.

Illinois State Board of Health, directory.................. 527
Illinois State Board of Health, examination of.............. 554
Illinois State Medical Society, proceedings of.............. 607
Impermeable plasters, effects on men........................ 426
Infra-orbital nerve, resection of. 214
Ingals, E. Fletcher, M. D., case of poisoning by chloroform.. 400
Ingals, E. Fletcher, M. D., danger from hypodermic injections of morphia......... 491
Instruments and apparatus, new 376
Intestine, carcinoma of, case... 346
Intestine, tuberculous ulceration of, case..................... 351
Intussusception cured by reduction.......................... 96
Iodine, colorless tincture of.... 603
Iodoform..................... 98
Iron, contra-indications in women....................... 415

J.

Jacobson, S. D., syphilitic lesions of nerve-centres....... 285
James, Martin L., M. D., heart clots....................... 535
Jaundice, treated by enemata of cold water................. 213
Jewell, J. S., M. D., review of Brown-Séquard's lectures ... 561
Joints, antiseptic injections in, Dr. Fr. Remie............. 435
Joints, permanent extension of, Dr. N. Senn................ 17

PAGE.
Jones, H. W., diabetes cured by skim milk.................. 31
Jones, M. O., treatment of vomiting of pregnancy.......... 630

K.

Kidneys, amyloid degeneration of, case.................... 342
Kidneys, cirrhosis of.......... 350
Kidney, encepholoid cancer of, case....................... 347
Knee joint, excision of......... 90
Knee joint, incised penetrating wounds of.................. 549

L.

Laparo-elytrotomy, Dr. T. G. Thomas..................... 540
Lee, E. W., M. D., tracheotomy, cases..................... 501
Lithotomy, a case............ 165
Lithotrity, cases of; an instrument for finding small fragments of stone, Dr. E. Andrews.................. 592
Liver, amyloid degeneration of, case...................... 342
Liver, cirrhosis of, case....... 350
Liver, indurating inflammation of........................ 339
Lower limbs, how to measure, Dr. H. Banga.............. 621
Lymphatic system, anatomy of. 279

M.

Maternal impressions and mother's marks, Dr. Roswell Park 522
Mattus, J. M., M. D., case by... 398
Meacher, Wm., M. D., ovariotomy...................... 598
Measurements of the extremities, Dr. John Bartlett....... 484
Medical activity in the U. S., evidence of in foreign journals 366
Medical charities, abuse of.... 606
Medical college commencements..................... 444
Medical news and items....... 104, 222, 444, 555, 669
Medical Society, Arkansas State, proceedings of............. 670
Medical Society, Illinois State, proceedings of............. 607
Mesocephalic origin of sensitive troubles, Dr. Couty......... 644
Metallotherapy............... 642
Michigan State board of health 556

PAGE.
Migraine thirty years standing cured by static electricity.... 72
Milk, intravenous injection of.. 640
Milk sickness in animals, Dr. S. S. Gray.................. 533
Merriman, D. J., M. D., letter from...................... 416
Molluscum contagiosum, Dr. Geo. H. Fox............... 466
Montgomery, W. T., report of 565 cases of eye diseases..... 38
Mother's marks, etc........... 522
Mucilage.................... 571

N.

Nairne, J. T., M. D., external use of tincture of belladonna in night sweating........... 606
Neck, presentation of, case. ... 246
Nephritis, acute disquamative.. 339
Nerve-centers, syphilitic lesion of...................... 285
Nerve-stretching, Prof. Nuss baum's operation........... 225

O.

Obstetrical Gazette............ 558
Obstetrical operation, remarks on........................ 257
Observations in practice, surgery, gynæcology and obstetrics.................... 245
Observations in practice, gynæcology and obstetrics....... 161
Oral abcess, 3 cases.......... 89
Olecranon and patella, treatment of transverse fracture of 86
Ovariotomy, case............. 44
Os tincæ, occlusion of, a case.. 168
Ovarian tumor reported cured by galvanic current......... 272
Ovariotomy, case of, Dr. William Meacher.................. 598
Ovariotomy, double........... 260

P.

Paralysis, convulsions, etc., Dr. Brown-Séquard............. 449
Paralysis and convulsions as effects of diseases of the base of the brain, Dr. Brown-Séquard. 353
Park, R., M. D............... 631
Park, Roswell, M. D., maternal impressions and mother's marks...................... 522
Parkinson's disease, the trembling in.................... 79

PAGE.
Paralysis and convulsions as effects of disease of the base of the brain...........291, 353, 449
Parotid gland, partial extirpation of..................... 277
Parrot, M., syphilis........... 631
Psoas abcess, diagnosis of...... 530
Patella and olecranon, treatment of the transverse fracture of.. 86
Patella, treatment of fracture of. 631
Pathological transactions of the Chicago Medical Society, Dr. I. N. Danforth............1, 337
Pauper practice, bidding for... 618
Peaslee, E. R., M. D., obituary. 443
Pelvis, contracted, cases....248, 255
Perineum, discussion on primary or secondary operation for ruptured................. 288
Perineum, how to save, Dr. E. Warren Sawyer.............. 496
Perityphlitic abcess, incisions.. 533
Pessary, a new stem, with movable disc, E. Cutter.......... 25
Phonograph.................. 669
Phthisis and tuberculosis, some observations on the clinical and pathological distinction between, Dr. W. T. Belfield.. 1
Phthisis, early signs of........ 74
Pilocarpin, action of.......... 218
Placenta prævia, cases......... 172
Placenta prævia, the prophylactic treatment of............. 85
Plummer, S. C., double ovariotomy...................... 260
Pneumonia, salycilic acid in.... 181
Pneumogastric nerves, abstract of paper on ................ 282
Pregnancy, vomiting of, treatment..................... 630
Poisoning by American dried beef...................... 391
Poisoning by chloroform, case. 398
Poisoning by custards and ice cream..................... 534
Poisoning by hypodermic use of morphia................. 491
Poison oak, ivy, etc., the remedy for eruption of............. 521
Poisoning by strychnine, recovery of damages............. 513
Pooley, J. H., M. D., perityphylitic abcess................ 533
Porrigo, pomade for........... 218
Pott's disease of vertebra, a new apparatus for, Dr. W. Blanchard....................... 376
Powell, G. P., M. D., glycerine in hæmorrhoids............. 642

PAGE.

Practice, private, notes from... 44, 179, 272, 396, 516, 604
Pregnancy, extra-uterine, cases, 113, 179
Pregnancy, extra-uterine during normal labor................ 84
Presentation, hand and cord, cases........................ 245
Prostrate, enlarged, cure of retention from................ 216
Purpura hæmorrhagica, pathology and treatment, Dr. N. S. Davis....................525, 585

Q.

Quinine exanthem............ 97
Quinine, hydrobromate of, in diseases of children, Dr. Steinitz......................... 438

R.

Ransom, W. L., M. D., cancer of stomach, etc................. 521
Rea, R. L., M. D.............. 426
Reviews: Burnett Charles H., the ear, its anatomy, physiolygy and diseases........... 64
Reviews and book notices:
Forensic medicine and toxicology, W. B. Woodman, M. D., etc., and C. M. Tidy, M. D., etc... ........... 626
Modern Organic Chemistry, Prof. C. G. Wheeler....... 625
Practical gynæcology, Dr. Heywood Smith; diseases of nasal cavity, etc., Dr. Carl Michel.................... 427
Handbook of practice of medicine, M. Charteris........ 431
The advantages and accidents of artificial anæsthesia, L. Turnbull, M. D............. 624
Resuscitation in poisoning and asphyxia............. 546
Transactions of Am. Dermatalogical Association...... 629
Reviews:
Ott, Isaac, action of medicine. 319
Corr, L. H., obstetrics reduced to questions and answers.. 320
Cerebral hyperæmia, Dr. Wm. A. Hammond, transactions of the N. Y. Pathological Society, injuries of the eye and their medico-legal aspect........................ 537
Durkee Silas, a treatise on gonorrhœa and syphilis... 205

PAGE.

Anstie, F. E., on use of wines in health and disease...... 322
Mitchell, S. W., nurse and patient, and camp cure...... 321
Public health reports....... 66
Spinal disease and spinal curvature, Lewis A. Sayre, M. D.................... 582
Transactions of State Medical Society of Penn.; transactions of State Medical Society of Ohio; transactions of State Medical Society of Virginia; transactions of Canada Medical Association..................... 530
Wylie, W. G., hospitals, their history, organization, and construction.............. 198
Ziemssen, Cyclopædia of Medicine, vol. XV........ 200
Ziemssen, Cyclopædia of Medicine, vol. XVI........ 203
Revulsive, new............... 439
Reynolds, Wm. E., M. D., value of electricity in paralysis.... 404
Richey, S. O., M. D., paper on exfoliation of cochlea....... 405
Rinne, Dr. Fr., M. D., antiseptic injections into joints........ 435
Rose, J. M., M. D., ruptures of uterus..................... 465
Rush Medical College, commencement................ 444

S.

Salicylate of soda for rheumatism, gout, etc............... 56
Salicylate of soda in diabetes.. 641
Salicylic acid in pneumonia... 181
Salisbury, J. H., M. D., arsenic in subnitrate of bismuth ... 601
Sawyer, E. Warren, M. D., how to save the perineum........ 496
Scott, T. A., M. D., superfœtation........................ 605
Senn, N., Dr., the treatment of inflammation of joints by permanent extension........... 17
Sexual orgasm, phenomena of, etc., Dr. J. Nevins Hyde..... 572
Shoulder-presentation, cases... 247
Sigmoid flexure, dilatation of, case....................... 400
Silvěrthorn, L. L., salicylic acid in pneumonia............... 181
Sloan, M. G., M. D., case of fibroid tumor of uterus cured by ergotine................. 604
Small pox, an ectrotic in...... 623

PAGE.
Smolt, C. F., M. D., case of cirrhosis of liver and kidneys 350
Society, Arkansas State Medical 527
Society, Chicago Medical, report ...............284, 413, 522
Society, Ill. State Medical..... 558
Society reports, Æsculapian Society, Wabash Valley..... 183
Society reports, the Brainard District Medical............ 52
Society of Physicians and Surgeons, report.......279, 405, 526
Society reports, Indiana, Illinois and Kentucky Tri-State Medical.......... ........... 49
Society reports, Medical Society District of Columbia....... 184
Sodic salicylate in diabetes ... 641
Spermatorrhœa, belladonna in. 274
Spine, dislocation of.......... 548
Spleen, case of abscess of, with perforation of large intestines, Dr. Geo. E. Brown.......... 1
Spleen, amyloid degeneration of, case ........ ........... 342
Staples, Franklin, gunshot wound of bladder, case...... 510
Stomach, cancer of, remarkable case ...................... 392
Stomach and liver, cancer of... 516
Stomach pump, modification of 392
Superfœtation or twin conception, Dr. T. A. Scott........ 605
Syphilis, origin of the term... 104
Syphilitic lesion of nerve centres...................... 285
Syphilis, innocuity of certain physiological secretions in... 145
Syphilis, by M. Parrot ....... 631

T.

Tayuya....................... 559
Tetanus, therapeutics of....... 441
Thomas, T. G., M. D., laparo-elytrotomy ................. 540
Thrombosis of coronary artery, case........................ 434
Thompson, Sir Henry's table of mortality from lithotomy.... 592
Tidd, Dr., chloral in retention of urine ................... 542
Tilley, R., M. D.,............. 631
Tincture of iodine, colorless... 603
Tongue, carcinoma of......... 47
Tracheotomy, cases........... 381
Tracheotomy in diphtheria.... 329
Tracheotomy in diphtheritic croup.................. ..... 121
Tracheotomy in diphtheria and croup, cases, Dr. E. W. Lee.. 501

PAGE.
Transfusion, Dr. M'Clintock... 543
Transfusion, a substitute for... 640
Tuberculous ulceration of intestine, case ................ 351
Twins, two heads, one body, living...................... 541
Typhoid fever, the bath in treatment of..................... 33

U.

University of London, admission of women................ 404
Uterus, inversion of, a case.... 171
Uterus, inertia, cases.......... 247
Urine, colored by carbolic acid. 643
Uterus, fibroid tumor of, treatment by ergotine............ 604
Urethritis provoked by arsenical preparations....... .... 102
Urethral fistula, elastic ligature in, Dr. J. H. Packard........ 533

V.

Vaccination, intra-uterine...... 72
Van Buren, Henry, death from æther and chloroform....... 268
Van Ripen, M. H., a case of extra-uterine pregnancy..... 179
Vaseline and salicylic acid in obstetrics ................. 551
Version, ano-pelvian.......... 83
Version, cases................. 171

W.

Walker, Geo. B., observations in practice..................... 245
Walker, G. B., observations in practice, etc.... ........... 161
Wellford, J. S., M. D., poisoning by custards, etc.............. 534
White, J. L., Hot Springs of Arkansas.................. 308
Whitmore, Jas. S., use of carbolic acid in treatment of erysipelas .... .............. 239
Willard, E. R., ovariotomy, case 44
Wilson, R. M., a case of diphtheria benefited by inhalation of steam ......... ...... 275
Women, admission of, to University of London..... ..... 404
Woman's Hospital Medical College, commencement........ 446
Wright, John, M. D., bidding for pauper practice ......... 618
Wright, J., bidding for pauper practice.... ................ 314

Y.

Yandell, L. P., M. D., obituary. 442

### BOOKS AND PAMPHLETS RECEIVED.

Sixth Annual Report of the Secretary of State of the State of Michigan; Relating to the Registry and Return of Births, Marriages and Deaths for the Year 1872.

Eulogy upon Lunsford P. Yandell, M. D. By Theodore S. Bell, M. D. Louisville, Ky. Reprint from *American Practitioner*, April, 1878.

Old Age, Its Diseases and Its Hygiene. By Lunsford P. Yandell, M. D., Louisville, Ky. Reprint from *American Practitioner*, February, 1878.

Typical Case of Addison's Disease, with Remarks. By George Ross, A. M., M. D. Professor Clinical Medicine, McGill University; Attending Physician Montreal General Hospital. Reported by Mr. H. N. Vineberg.

The Paralysis of Potts' Disease; Being a Clinical Study of Fifty-eight Cases. By V. P. Gibney, A. M., M. D., Assistant Surgeon to the Hospital for the Ruptured and Crippled. New York. Reprint from *Journal of Nervous and Medical Diseases*, 1878.

Transactions of the South Carolina Medical Association; 27th Annual Session, held in Charleston, S. C., April 10th and 11th, 1877.

Prescription Writing, Designed for the Use of Medical Students Who Have Never Studied Latin. By Frederic Henry Gerrish, M. D., Professor of Materia Medica and Therapeutics in the Medical School of Maine, etc Second edition. Pub. Portland, Me.: Loring, Short & Harmon. Philadelphia: J. B. Lippencott & Co.

---

THE *London Lancet* for May, 1878, contains an interesting communication from Dr. M. O. Jones, of Chicago, on the treatment of the vomiting of pregnancy, to which is added a note of a case by Dr. Marion Sims, who considers Dr. Jones' method of treatment important and worthy of more extended trial. It consists in pencilling the os uteri with the solid nitrate of silver. Usually but one application has been found to be necessary, and the gratifying relief which followed was obtained within twenty-four hours after the application.

Dr. Jones is to be congratulated on having his procedure introduced to the profession with such high endorsement.

### ANNOUNCEMENTS FOR THE MONTH.

### SOCIETY MEETINGS.

Chicago Medical Society—Mondays, June 10 and 24.

Chicago Society of Physicians and Surgeons—Mondays, June 3 and 17.

### CLINICS.

MONDAY.

Eye and Ear Infirmary—2 to 4 p. m., by Prof. Holmes and Dr. Hotz—2 p. m., Prof. Jones.

Mercy Hospital—2 to 3 p. m. Surgical, by Prof. Andrews.

Rush Medical College—2:30 p. m. Dermatological and Venereal, by Dr. Hyde.

County Hospital—8 p. m. Necropsy, by Dr. Danforth.

Woman's Medical College—3 p. m. Surgical, by Prof. Owens.

TUESDAY.

County Hospital—1:30 p. m. Medical, by Prof. Lyman; 2:30 p. m. Surgical, by Prof. Parkes.

Mercy Hospital—2 p. m. Medical, by Prof. Hollister.

Eye and Ear Infirmary—2 p. m. Prof. Jones.

WEDNESDAY.

County Hospital—2 p. m. Gynecological, by Dr. Bridge. 3 p. m. Ophthalmological, by Dr. Montgomery.

Mercy Hospital—2 p. m. Eye and Ear, by Prof. Jones.

Rush Medical College—4 p. m. Diseases of the Chest, by Dr. E. Fletcher Ingals.

THURSDAY.

Mercy Hospital—2 p. m. Medical, by Prof. Davis.

Rush Medical College—1:30 p. m. Neurological, by Prof. Lyman.

Eye and Ear Infirmary—2 to 4 p. m. Operations by Prof. Holmes and Dr. Hotz.

FRIDAY.

Mercy Hospital—2 p. m. Medical, by Prof. Davis.

County Hospital—1:30 p. m. Medical, by Prof. Quine; 2:30 p. m., Surgical, by Prof. Powell.

Woman's Medical College—10 p. m. Ophthalmological, by Dr. Montgomery.

SATURDAY.

Rush Medical College—2 p. m. Surgical, Prof. Gunn.

Chicago Medical College—2 p. m. Surgical, by Prof. Andrews and Isham; 3 p. m., Diseases of the Chest, by Prof. Johnson.

Woman's Medical College—12 m. Gynecological, by Prof. Fitch; 3 p. m. Dermatological, Dr. Maynard.

Special Clinics daily, from 2 to 4 p. m., at the South Side Dispensary, and at the Centrl Free Dispensary.

For schedule of lectures at the colleges, apply to the college janitors.

